# Mitochondrial Haplogroups and Control Region Polymorphisms in Age-Related Macular Degeneration: A Case-Control Study

**DOI:** 10.1371/journal.pone.0030874

**Published:** 2012-02-13

**Authors:** Edith E. Mueller, Elena Schaier, Susanne M. Brunner, Waltraud Eder, Johannes A. Mayr, Stefan F. Egger, Christian Nischler, Hannes Oberkofler, Herbert A. Reitsamer, Wolfgang Patsch, Wolfgang Sperl, Barbara Kofler

**Affiliations:** 1 Research Program for Receptor Biochemistry and Tumor Metabolism, Department of Pediatrics, Paracelsus Medical University, Salzburg, Austria; 2 Department of Ophthalmology, Paracelsus Medical University, Salzburg, Austria; 3 Department of Laboratory Medicine, Paracelsus Medical University, Salzburg, Austria; Washington University School of Medicine, United States of America

## Abstract

**Background:**

Onset and development of the multifactorial disease age-related macular degeneration (AMD) are highly interrelated with mitochondrial functions such as energy production and free radical turnover. Mitochondrial dysfunction and overproduction of reactive oxygen species may contribute to destruction of the retinal pigment epithelium, retinal atrophy and choroidal neovascularization, leading to AMD. Consequently, polymorphisms of the mitochondrial genome (mtDNA) are postulated to be susceptibility factors for this disease. Previous studies from Australia and the United States detected associations of mitochondrial haplogroups with AMD. The aim of the present study was to test these associations in Middle European Caucasians.

**Methodology/Principal Findings:**

Mitochondrial haplogroups (combinations of mtDNA polymorphisms) and mitochondrial CR polymorphisms were analyzed in 200 patients with wet AMD (choroidal neovascularization, CNV), in 66 patients with dry AMD, and in 385 controls from Austria by means of multiplex primer extension analysis and sequencing, respectively. In patients with CNV, haplogroup H was found to be significantly less frequent compared to controls, and haplogroup J showed a trend toward a higher frequency compared to controls. Five CR polymorphisms were found to differ significantly in the two study populations compared to controls, and all, except one (T152C), are linked to those haplogroups.

**Conclusions/Significance:**

It can be concluded that haplogroup J is a risk factor for AMD, whereas haplogroup H seems to be protective for AMD.

## Introduction

In the western world, age-related macular degeneration (AMD) is the most frequent cause of visual loss in people aged 50 or older [1]. The incidence of AMD is low in persons younger than 50 years (0.05%), but climbs to 11.8% in persons over the age of 80 [2]. AMD affects the macula, a retinal region containing the highest density of photoreceptors and generating central high-resolution visual acuity. In AMD, the presence of so-called drusen, deposits of acellular debris in between the retinal pigment epithelium and the Bruch's membrane, localized posterior to the photoreceptors, is the first characteristic sign of the disease. A few small drusen may appear in the retina of individuals over 50 years of age without relation to AMD. An excess of drusen and/or medium sized or large drusen, however, may lead to destruction of the retinal pigment epithelium and, in combination with inflammatory processes, cause atrophy of the retina and mild visual impairment. Subsequent retinal atrophy reaching further into the center of the macula (dry AMD), and also choroidal neovascularization (CNV, wet AMD) with augmented vascular permeability and fragility resulting in retinal edema and/or hemorrhage, may cause visual loss [1].

AMD is a multifactorial disease, where several risk factors and genetic variants may act together, resulting in disease development. Besides age, known risk factors are amongst others smoking and white race [1]. Nuclear encoded genetic susceptibility factors for AMD include the polymorphism Tyr402His in the complement factor H (*CFH*) gene, as well as the Ala69Ser polymorphism in the age-related maculopathy susceptibility 2 gene (*ARMS2*) [1], [3], [4].

The retinal pigment epithelium, being metabolically very active, comprises a high number of mitochondria [5]. In a process called oxidative phosphorylation (OXPHOS), these organelles produce most of the cellular energy in the form of ATP, as well as reactive oxygen species (ROS). Thirteen subunits of the OXPHOS enzymes are encoded by the mitochondrial genome (mtDNA) [6]. ROS, including those produced in mitochondria by the electron transport chain, preferentially damage mtDNA. In turn, damaged mtDNA induces mitochondrial dysfunction with disturbed OXPHOS and higher production of ROS, initiating a vicious circle [7].

The retinal pigment epithelium is especially susceptible to mitochondrial dysfunction and ROS damage and, being a post-mitotic tissue, does not regenerate, and thus accumulates mtDNA somatic mutations [8], [9]. A higher prevalence of AMD in older people and the progressive nature of the disease, as well as the high susceptibility of the retina to oxidative stress, hint at mitochondrial involvement in the progress of AMD.

Interestingly, some patients suffering from mitochondrial diseases caused by mutations or deletions of mtDNA, such as Leber's hereditary optic neuropathy (LHON) or Kearns-Sayre syndrome, have been shown to exhibit retinal pigmentary changes resembling features of the early AMD phenotype [10], [11].

Neutral polymorphisms of mtDNA or combinations of these, defined as mtDNA haplogroups, might also contribute to AMD onset or progression. Mitochondrial haplogroups (H, J, U and T) and polymorphisms (73, 4917 and 16126, among others) have already been detected to be associated with AMD in case-control studies from the United States (US) and Australia [12]–[15]. In epidemiological studies it is important to obtain repetitive results in different studies and different geographical regions. This is of high relevance for interpretation of such data, in order to avoid the influence of possible confounding factors that might bias the result of one particular study. In studies analyzing mtDNA, repetitive results are even more crucial, as haplogroup distributions can vary between populations within only small regional distances [16].

Hence, the aim of the present study was to confirm associations between mtDNA haplogroups and AMD that were obtained in US and Australian populations, in Middle-European Caucasians.

## Results

### Mitochondrial haplogroups H and J are associated with CNV

In the present case-control study, haplogroups and polymorphisms of the non-coding CR of mtDNA were analyzed in patients with wet AMD (CNV) and dry AMD and compared to those in controls. Clinical characteristics of the study groups are presented in Table 1.

**Table 1:** Characteristics of the study populations.

	Patients with CNV^a^	Patients with dry AMD^b^	Control group
	n = 200	n = 66	n = 385
Mean (SD^c^) age (years) at diagnosis	77.2 (8.4)	80.9 (6.0)	73.6 (9.5)
Male (%)	34.0	42.4	49.9

a CNV = choroidal neovascularization.

b AMD = age-related macular degeneration.

c SD = standard deviation.

The frequencies of mitochondrial haplogroups and CR polymorphisms in the control group were very similar to those previously reported in a large control group from Salzburg [17]–[20].

In patients with CNV, haplogroup H was found at a significantly lower frequency than in the control group [36.0% vs. 44.9%, p = 0.038, OR 0.69 (0.5–1.0); after adjustment for age and sex: p = 0.035, OR 0.68 (0.5–1.0)] (Table 2).

**Table 2:** Frequencies (%) of mitochondrial haplogroups in Caucasian patients with CNV and in controls.

	Patients with CNV^a^	Control group	P-value^b^
	n = 200	n = 385	
H	36.0	44.9	0.038
U	18.0	15.9	0.506
J	15.0	9.6	0.052
T	10.5	7.5	0.223
K	5.5	3.9	0.372
W	0.5	2.3	0.176
V	2.0	3.4	0.347
I	2.0	0.8	0.238
X	2.0	1.6	0.742
Others^c^	8.5	10.1	0.525

a CNV = choroidal neovascularization.

b P-value: Pearson chi-square or Fisher's exact test.

c Haplogroups that could not be assigned to one of the nine major European haplogroups by the SNP combination.

Moreover, haplogroup J was more frequent in patients with CNV compared to the control group [15.0% vs. 9.6%, p = 0.052, OR 1.66 (1.0–2.8); after adjustment for age and sex: p = 0.048, OR 1.72 (1.0–2.9)] (Table 2).

When haplogroup frequencies were compared between patients with dry AMD and the controls, no significant differences were found (Table 3).

**Table 3:** Frequencies (%) of mitochondrial haplogroups in Caucasian patients with dry AMD and in controls.

	Patients with dry AMD^a^	Control group	P-value^b^
	n = 66	n = 385	
H	48.5	44.9	0.593
U	13.6	15.9	0.647
J	7.6	9.6	0.599
T	9.1	7.5	0.662
K	3.0	3.9	1.000
W	6.1	2.3	0.107
V	3.0	3.4	1.000
I	0.0	0.8	1.000
X	4.5	1.6	0.131
Others^c^	4.6	10.1	0.149

a AMD = age-related macular degeneration.

b P-value: Pearson chi-square or Fisher's exact test.

c Haplogroups that could not be assigned to one of the nine major European haplogroups by the SNP combination.

### Age-related macular degeneration and mitochondrial CR polymorphisms

The mitochondrial CR was sequenced and analyzed between nucleotide position 16038 and 569. All polymorphisms and their frequencies and the results of comparisons between patients with CNV and controls as well as between patients with dry AMD and controls are listed in Table S1 and Table S2, respectively. CR polymorphisms with a frequency higher than 5% are listed in Table 4 and Table 5. The CR polymorphism T152C was found to be present at a significantly higher frequency in patients with CNV compared to controls. An additional four CR polymorphisms were significantly more frequent in patients with CNV compared to controls (Table 4). However, three of these are linked to mitochondrial haplogroup J [C16069T (J), T16126C (JT), C295T (J)] [21]. The mtDNA CR position 73A defines the macro-haplogroup R0, including its predominant subclade haplogroup H as well as haplogroup V [21], [22]. The CR polymorphism A73G, was found to have a significantly higher frequency in CNV patients compared to controls. Because of the inverse correlation of A73G to haplogroup H, the results of the mitochondrial CR polymorphism analysis are consistent with the results of mitochondrial haplogroup frequencies.

**Table 4:** Frequencies (%) of control region polymorphisms >5% in patients with CNV and in controls as well as the corresponding odds ratios and 95% confidence intervals.

Polymorphism in mtDNA^a^ control region	Frequency in patients with CNV^b^	n^c^	Frequency incontrol group	n^c^	P-value^d^	Odds ratio(95% CI^e^)	P-value^f^	aOdds ratio(95% CI^e^)^f^
	n = 200		n = 385					
C16069T	16.00	32	10.13	39	0.039	1.69 (1.0–2.8)	0.038	1.74 (1.0–2.9)
T16093C	9.00	18	8.31	32	0.778			
T16126C	28.50	57	18.70	72	0.007	1.73 (1.2–2.6)	0.006	1.79 (1.2–2.7)
T16189C	14.00	28	11.17	43	0.320			
C16223T	6.50	13	7.01	27	0.816			
C16270T	9.50	19	5.71	22	0.089			
C16294T	11.50	23	9.61	37	0.475			
C16296T	6.00	12	6.49	25	0.816			
T16304C	8.50	17	9.87	38	0.590			
T16311C	14.00	28	9.87	38	0.134			
T16356C	5.50	11	7.27	28	0.415			
T16362C	7.50	15	6.49	25	0.647			
T16519C	63.00	126	65.45	252	0.556			
A73G	62.50	125	51.43	198	0.011	1.57 (1.1–2.2)	0.012	1.58 (1.1–2.3)
T146C	12.50	25	8.05	31	0.083			
C150T	12.50	25	10.13	39	0.384			
T152C	28.50	57	20.78	80	0.036	1.52 (1.0–2.3)	0.014	1.67 (1.1–2.5)
G185A	8.00	16	5.71	22	0.287			
T195C	18.50	37	17.92	69	0.863			
G228A	8.50	17	5.97	23	0.251			
A263G	98.50	197	98.44	379	1.000			
C295T	16.50	33	10.39	40	0.034	1.70 (1.0–2.8)	0.035	1.74 (1.0–2.9)
A302InsC	43.50	87	36.62	141	0.106			
A302InsCC	9.00	18	13.25	51	0.131			
T310InsC	94.50	189	94.03	362	0.816			
C462T	11.00	22	6.75	26	0.076			
T489C	16.50	33	10.91	42	0.055			
CA514/515Del	10.50	21	10.13	39	0.889			

a mtDNA = mitochondrial DNA.

b CNV = choroidal neovascularisation.

c n: number of individuals with the respective polymorphism.

d P-value: Pearson chi-square or Fisher's exact test.

e CI = confidence interval.

f adjusted for age and sex by logistic regression analysis.

**Table 5:** Frequencies (%) of control region polymorphisms >5% in patients with dry AMD and in controls.

Polymorphism in mtDNA^a^ control region	Frequency in patients with dry AMD^b^	n^c^	Frequency incontrol group	n^c^	P-value^d^
	n = 66		n = 385		
C16069T	9.09	6	10.13	39	0.795
T16093C	7.58	5	8.31	32	0.840
T16126C	16.67	11	18.70	72	0.694
T16189C	13.64	9	11.17	43	0.562
C16223T	12.12	8	7.01	27	0.152
C16294T	9.09	6	9.61	37	0.894
T16298C	6.06	4	5.97	23	1.000
T16304C	9.09	6	9.87	38	0.844
T16311C	12.12	8	9.87	38	0.577
T16356C	7.58	5	7.27	28	1.000
T16362C	6.06	4	6.49	25	1.000
T16519C	75.76	50	65.45	252	0.100
A73G	54.55	36	51.43	198	0.640
T146C	7.58	5	8.05	31	0.895
C150T	12.12	8	10.13	39	0.625
T152C	24.24	16	20.78	80	0.525
T195C	33.33	22	17.92	69	0.004
A263G	100.00	66	98.44	379	0.599
C295T	7.58	5	10.39	40	0.481
A302InsC	30.30	20	36.62	141	0.322
A302InsCC	10.61	7	13.25	51	0.554
T310InsC	93.94	62	94.03	362	1.000
T489C	7.58	5	10.91	42	0.413
G499A	7.58	5	6.23	24	0.596
G513InsCA	6.06	4	5.97	23	1.000
CA514/515Del	7.58	5	10.13	39	0.518

a mtDNA = mitochondrial DNA.

b AMD = age-related macular degeneration.

c n: number of individuals with the respective polymorphism.

d P-value: Pearson chi-square or Fisher's exact test.

Twenty-six CR polymorphisms were found with a frequency higher than 5% in both controls and patients with dry AMD. Of these, T195C had a significantly higher frequency in patients with dry AMD compared to controls [33.3% vs. 17.9%, p<0.005, OR 2.29 (1.3–4.1); after adjustment for age and sex: p<0.01, OR 2.30 (1.3–4.2)] (Table 5).

## Discussion

Studies have shown that oxidative stress plays a causative role in the pathogenesis of AMD, and that mitochondria are instrumental in this etiology. For example, Imamura et al. observed in the retinas of *Sod1^−/−^* mice, which lack the antioxidant enzyme Cu, Zn-superoxide dismutase (SOD1), elevated levels of oxidative damage to DNA and protein as well as development of drusen and CNV with age, signs shared by human AMD [23]. Vives-Bauza et al. reported that a combination of mild OXPHOS defects, caused by the mtDNA T8993G point mutation, together with low levels of the autofluorescent constituent of lipofuscin, A2E, induced a reduction in the phagocytic competence of the retinal pigment epithelium. The combination of low levels of A2E and more severe OXPHOS defects induced retinal pigment epithelium cell death [9]. All these facts support the conclusion that mtDNA variation contributes to the complex etiology of AMD.

Accordingly, in the present study we observed a higher prevalence of mitochondrial haplogroup J and a significantly lower prevalence of haplogroup H in patients with CNV compared to the control group. That the CR polymorphisms linked to haplogroups J and H also showed positive and negative associations, respectively, with CNV further supports the haplogroup–CNV association.

The CR polymorphism T152C, although it is not associated with any particular haplogroup, was also present at a higher frequency in patients with CNV compared to controls. For dry AMD, only one significant association was found, namely a higher frequency of T195C in patients compared to controls. The study group of patients with dry AMD (n = 66) was small, and also no data were available according to a classification into early, intermediate or advanced AMD. Hence, these results should be considered tentative. Associations of T152C and T195C have been reported, with breast cancer in Tunisian women (T152C – weak protective effect) [24] and with early childhood bronchitis (T195C – increased risk) [25]. The mitochondrial CR polymorphisms T152C and T195C are mutational hotspots [21], [26], and are found on the background of several haplogroups.

Lack of Bonferroni correction might be considered to be a limitation of the present study. However, we believe that Bonferroni correction is not the adequate method for our type of analysis, because our study was intended to be a confirmation study and because CR polymorphisms that showed significantly different frequencies between controls and patients are strongly linked to haplogroups H and J.

Jones et al. compared mtDNA haplogroup frequencies in 317 patients with AMD to those of 2985 controls from Australia. After adjustment for age, sex and smoking they found haplogroup H to be at a reduced prevalence in the AMD group compared to controls, very similar to the results obtained in the present study (Table 6). Additionally, subjects with haplogroup J were found to have a higher risk of developing large, soft, distinct drusen [12]. Unfortunately, in our study no data concerning drusen development were available.

**Table 6:** Comparison of age-related macular degeneration case-control studies in the literature with the present study.

MtDNA^a^ haplogroup or polymorphism	Haplogroup^b^	Frequency (%) in cases	Frequency (%) in controls	P-value^c^	Odds ratio (95% CI^d^)	References
H		37.5	43.5	not shown	0.75 (0.6–1.0)	[12]
		36.0	44.9	0.035	0.68 (0.5–1.0)	Present study
4917G	T	15.4	9.0	0.011	2.16 (1.2–3.9)	[13]
		12.2	2.7	0.020	5.00 (1.1–23.6)	[14]
		16.3	4.0	0.001	6.15 (2.0–18.5)	[15]
		11.5	8.3	0.143	1.54 (0.9–2.8)	Present study
T		10.5	7.5	0.133	1.59 (0.9–2.9)	Present study
73G	non-HV,H,V	62.5	51.4	0.012	1.58 (1.1–2.3)	Present study
73A	HV,H,V	35.8	48.5	0.030	0.58 (0.4–1.0)	[15]
16069T	J	9.9	4.1	0.055	3.89 (0.8–19.0)	[14]
		16.0	10.1	0.038	1.74 (1.0–2.9)	Present study
16126C	JT	24.7	8.2	0.004	3.66 (1.4–9.7)	[14]
		27.0	13.1	0.007	2.51 (1.3–4.9)	[15]
		28.5	18.7	0.006	1.79 (1.2–2.7)	Present study

a mtDNA = mitochondrial DNA.

b According to PhyloTree.org [21].

c P-values: present study: adjusted for age and sex. Jones et al. [12]: adjusted for age, sex and current smoking. Canter et al. [13]: adjusted for sex and three nuclear polymorphisms (CFH-Complement Factor H gene, rs1061170; LOC387715, rs10490924; APOE, ApoE2 allele). Udar et al. [14]: no adjustment. SanGiovanni et al. [15]: adjusted for age, sex and smoking.

d CI = confidence interval.

In 560 Caucasians from the US, Canter et al. detected the mitochondrial haplogroup T-linked polymorphism A4917G to be significantly associated with AMD (Table 6) [13]. In our study, we were not able to find a significant association with this polymorphism, although we did observe a tendency toward a higher frequency of A4917G (11.5% vs. 8.3%, data not shown) as well as of mitochondrial haplogroup T (10.5% vs. 7.5%) in patients with CNV compared to controls (Table 2 and Table 6).

A4917G and the CR polymorphisms C16069T and T16126C, which are linked to haplogroup J (C16069T) and the haplogroup cluster JT (T16126C), were found to be associated with AMD in a case-control study (73 controls and 81 patients) from the US (Table 6) [14]. Moreover, SanGiovanni et al. found a significant association of A4917G and the CR positions 73A and T16126C with advanced AMD in 314 (215 cases and 99 controls) non-Hispanic US and Australian subjects (Table 6) [15].

Our study adds to these previous studies from the US and Australia in providing corroborative data for a population from a geographically well-defined and distinct region of Middle Europe. Combining all the results, the conclusion can be drawn that mtDNA haplogroups J and T are risk factors, and that mtDNA haplogroup H is a protective factor, for AMD in Caucasian populations.

## Methods

### Ethics Statement

Before entering the study, subjects gave written informed consent, and anonymity of the patients was assured. The study was performed in accordance with the National Gene Technology Act of Austria and followed the Declaration of Helsinki. The study was approved by the Local Province of Salzburg Ethics Committee (“Ethikkommission für das Bundesland Salzburg; Amt der Salzburger Landesregierung, Abteilung 9 Gesundheit und Sport”).

### Patients and control subjects

In this case-control study we included a total of 651 Caucasian subjects. All participants were enrolled in the Department of Ophthalmology, University Hospital Salzburg, Paracelsus Medical University. Blood samples from patients and controls were collected from August 2006 to October 2008. Recruitment of patients and controls was done as follows. Each patient diagnosed for CNV was asked if he agreed to participate in the study. Controls were mainly subjects who were kept as an inpatient for cataract surgery. Here, subjects were asked for agreement sporadically. Patients with dry AMD were also recruited sporadically. Sixty-six patients were diagnosed with dry AMD, 200 patients with CNV, and a control group of 385 subjects did not show any signs of AMD (i.e. no drusen, atrophy or pigment epithelium changes). Patients with secondary CNV resulting from myopia, inflammatory or infectious chorioretinitis, angioid streaks, hereditary diseases or trauma were not included in the present study.

In all participants a complete ophthalmological examination, including dilated fundus examination, was performed. Distance visual acuity was measured at a distance of 4 m using Snellen charts. When CNV was suspected, additionally fundus photography, fluorescein angiography and optical coherence tomography (OCT) were conducted. Fundus photography and fluorescence angiograms were obtained digitally with a Zeiss fundus camera and imaging software (FODAS), and central macular thickness was measured with a Zeiss-Humphrey OCT Stratus 3000 (Jena, Germany).

### Mitochondrial DNA analysis

Mitochondrial haplogroups were assessed as described before [27]. Haplogroups that could not be assigned to one of the nine major European haplogroups by their single nucleotide polymorphism (SNP) combination were designated as “others”.

CR sequences were analyzed between nucleotide positions 16038 and 569. Polymerase chain reaction and sequencing was performed as described previously [27]; however, the primer 15997f: 5′-CACCATTAGCACCCAAAGCT-3′ was used instead of 16098f.

### Statistical analysis

Frequencies of all mitochondrial haplogroups and CR polymorphisms were tested for independence for the disease using Pearson chi-square statistics and Fisher's exact test as appropriate. Only haplogroups and polymorphisms with a frequency higher than 5% in both study groups were subjected to further statistical analysis. Associations were adjusted for age and sex using logistic regression analysis. A p-value <0.05 was considered statistically significant. All analyses were performed using PASW statistics 18.0 (SPSS GmbH, Germany).

## Supporting Information

Table S1 **Frequencies (%) of control region (CR) polymorphisms in patients with CNV and in controls as well as the corresponding odds ratios and 95% confidence intervals.**
(DOC)

Table S2 **Frequencies (%) of control region (CR) polymorphisms in patients with dry AMD and in controls as well as the corresponding odds ratios and 95% confidence intervals.**
(DOC)
