# Supplementary material for: Mitochondrial Haplogroups and Control Region Polymorphisms in Age-Related Macular Degeneration: A Case-Control Study
Source: PLoS One. 2012 Feb 13;7(2):e30874. doi: 10.1371/journal.pone.0030874 (PMC3278404; doi:10.1371/journal.pone.0030874)
Supplement: Table S1 — Frequencies (%) of control region (CR) polymorphisms in patients with CNV and in controls as well as the corresponding odds ratios and 95% confidence intervals. (DOC) [file pone.0030874.s001.doc]

**Table S1.** Frequencies (%) of control region (CR) polymorphisms in patients with CNV and in controls as well as the corresponding odds ratios and 95% confidence intervals.

| Polymorphism in mtDNA^a^ control region | Frequency in patients with CNV^b^ | n^c^ | Frequency in control group | n^c^ | P-value^d^ | Odds ratio (95% CI^e^) |
| --- | --- | --- | --- | --- | --- | --- |
|  | n=200 |  | n=385 |  |  |  |
| T16045C^f^ | 0.00 | 0 | 0.26 | 1 | 1.000 |  |
| A16051G | 2.00 | 4 | 1.82 | 7 | 1.000 |  |
| T16064A^f^ | 0.50 | 1 | 0.00 | 0 | 0.342 |  |
| A16066G | 0.50 | 1 | 0.00 | 0 | 0.342 |  |
| C16067T | 0.50 | 1 | 0.52 | 2 | 1.000 |  |
| C16069T | 16.00 | 32 | 10.13 | 39 | 0.039 | 1.69 (1.0-2.8) |
| C16072T | 0.00 | 0 | 0.26 | 1 | 1.000 |  |
| A16080G | 0.00 | 0 | 0.52 | 2 | 0.549 |  |
| T16086C | 0.00 | 0 | 0.78 | 3 | 0.555 |  |
| T16086A^f^ | 0.50 | 1 | 0.00 | 0 | 0.342 |  |
| T16092C | 1.50 | 3 | 0.52 | 2 | 0.344 |  |
| T16093C | 9.00 | 18 | 8.31 | 32 | 0.778 |  |
| C16111T | 0.00 | 0 | 0.52 | 2 | 0.549 |  |
| C16111A | 0.50 | 1 | 0.00 | 0 | 0.342 |  |
| C16114A | 0.00 | 0 | 0.26 | 1 | 1.000 |  |
| T16124C | 1.00 | 2 | 0.78 | 3 | 1.000 |  |
| T16126C | 28.50 | 57 | 18.70 | 72 | 0.007 | 1.73 (1.2-2.6) |
| G16129A | 5.00 | 10 | 4.68 | 18 | 0.861 |  |
| G16129C | 1.00 | 2 | 0.78 | 3 | 1.000 |  |
| T16131C | 0.50 | 1 | 0.00 | 0 | 0.342 |  |
| C16134T | 0.00 | 0 | 0.78 | 3 | 0.555 |  |
| G16145A | 8.00 | 16 | 4.16 | 16 | 0.052 |  |
| A16146G | 0.50 | 1 | 0.00 | 0 | 0.342 |  |
| C16148T | 0.00 | 0 | 1.04 | 4 | 0.305 |  |
| G16153A | 2.00 | 4 | 0.26 | 1 | 0.049 | 7.84 (0.9-70.6) |
| T16157C | 0.50 | 1 | 0.00 | 0 | 0.342 |  |
| A16162G | 1.50 | 3 | 3.38 | 13 | 0.187 |  |
| A16162C | 0.00 | 0 | 0.26 | 1 | 1.000 |  |
| A16163G | 2.50 | 5 | 1.04 | 4 | 0.286 |  |
| A16165G | 0.50 | 1 | 0.00 | 0 | 0.342 |  |
| A16166G | 0.00 | 0 | 0.26 | 1 | 1.000 |  |
| C16167T | 0.50 | 1 | 0.00 | 0 | 0.342 |  |
| C16168T | 0.00 | 0 | 0.26 | 1 | 1.000 |  |
| C16169T | 1.00 | 2 | 1.04 | 4 | 1.000 |  |
| A16170G | 0.00 | 0 | 0.26 | 1 | 1.000 |  |
| T16172C | 4.00 | 8 | 1.04 | 4 | 0.027 | 3.97 (1.2-13.3) |
| C16173T | 0.00 | 0 | 0.26 | 1 | 1.000 |  |
| C16174T | 0.50 | 1 | 0.26 | 1 | 1.000 |  |
| C16176G | 0.00 | 0 | 0.26 | 1 | 1.000 |  |
| C16179T | 0.00 | 0 | 3.64 | 14 | 0.003 |  |
| A16180C | 0.50 | 1 | 0.00 | 0 | 0.342 |  |
| A16181C | 0.50 | 1 | 0.00 | 0 | 0.342 |  |
| A16182G | 0.00 | 0 | 0.26 | 1 | 1.000 |  |
| A16182C | 1.50 | 3 | 2.08 | 8 | 0.757 |  |
| A16183C | 5.00 | 10 | 3.90 | 15 | 0.531 |  |
| C16184T | 0.50 | 1 | 0.52 | 2 | 1.000 |  |
| C16185T | 0.00 | 0 | 0.26 | 1 | 1.000 |  |
| C16186T | 2.50 | 5 | 1.04 | 4 | 0.286 |  |
| C16187T | 0.50 | 1 | 0.00 | 0 | 0.342 |  |
| C16188T | 0.50 | 1 | 0.52 | 2 | 1.000 |  |
| T16189A | 0.00 | 0 | 0.26 | 1 | 1.000 |  |
| T16189C | 14.00 | 28 | 11.17 | 43 | 0.320 |  |
| T16189InsC | 0.50 | 1 | 0.00 | 0 | 0.342 |  |
| C16192T | 6.50 | 13 | 4.16 | 16 | 0.215 |  |
| C16193T | 2.00 | 4 | 1.56 | 6 | 0.742 |  |
| C16201T | 0.50 | 1 | 0.52 | 2 | 1.000 |  |
| A16207G | 1.00 | 2 | 0.26 | 1 | 0.270 |  |
| T16209C | 0.50 | 1 | 0.00 | 0 | 0.342 |  |
| A16212G | 0.00 | 0 | 0.78 | 3 | 0.555 |  |
| G16213A | 0.00 | 0 | 0.78 | 3 | 0.555 |  |
| A16216G | 0.50 | 1 | 0.00 | 0 | 0.342 |  |
| C16218T | 0.50 | 1 | 0.26 | 1 | 1.000 |  |
| C16221T | 0.50 | 1 | 0.78 | 3 | 1.000 |  |
| C16222T | 4.00 | 8 | 1.30 | 5 | 0.071 |  |
| C16223T | 6.50 | 13 | 7.01 | 27 | 0.816 |  |
| T16224C | 6.50 | 13 | 4.94 | 19 | 0.430 |  |
| T16231C | 4.50 | 9 | 3.12 | 12 | 0.394 |  |
| C16234T | 1.00 | 2 | 1.30 | 5 | 1.000 |  |
| A16235G | 0.50 | 1 | 0.52 | 2 | 1.000 |  |
| C16239T | 0.50 | 1 | 1.04 | 4 | 0.665 |  |
| C16242T | 0.00 | 0 | 0.52 | 2 | 0.549 |  |
| T16243C | 0.00 | 0 | 0.26 | 1 | 1.000 |  |
| G16244A | 0.00 | 0 | 0.26 | 1 | 1.000 |  |
| C16245T | 0.00 | 0 | 0.26 | 1 | 1.000 |  |
| C16248T | 1.00 | 2 | 0.26 | 1 | 0.270 |  |
| T16249C | 0.00 | 0 | 1.04 | 4 | 0.305 |  |
| G16255A | 1.50 | 3 | 1.04 | 4 | 0.695 |  |
| G16255Del^f^ | 0.50 | 1 | 0.00 | 0 | 0.342 |  |
| C16256T | 8.00 | 16 | 4.94 | 19 | 0.138 |  |
| A16258T | 0.00 | 0 | 0.26 | 1 | 1.000 |  |
| A16258C | 1.00 | 2 | 1.56 | 6 | 0.722 |  |
| C16259T | 0.50 | 1 | 0.00 | 0 | 0.342 |  |
| C16261T | 8.50 | 17 | 3.90 | 15 | 0.020 | 2.29 (1.1-4.7) |
| T16263C | 1.00 | 2 | 0.78 | 3 | 1.000 |  |
| C16264T | 0.50 | 1 | 0.00 | 0 | 0.342 |  |
| A16265G | 0.50 | 1 | 1.04 | 4 | 0.665 |  |
| A16265C | 0.50 | 1 | 0.00 | 0 | 0.342 |  |
| A16265T | 0.50 | 1 | 0.00 | 0 | 0.342 |  |
| C16266T | 0.00 | 0 | 0.52 | 2 | 0.549 |  |
| C16268T | 0.00 | 0 | 0.26 | 1 | 1.000 |  |
| A16269G | 0.00 | 0 | 0.26 | 1 | 1.000 |  |
| C16270T | 9.50 | 19 | 5.71 | 22 | 0.089 |  |
| T16271C | 1.00 | 2 | 0.78 | 3 | 1.000 |  |
| G16274A | 1.00 | 2 | 0.78 | 3 | 1.000 |  |
| C16278T | 4.00 | 8 | 3.12 | 12 | 0.577 |  |
| A16283T | 0.00 | 0 | 0.26 | 1 | 1.000 |  |
| A16284G | 0.50 | 1 | 0.00 | 0 | 0.342 |  |
| C16287T | 1.50 | 3 | 0.78 | 3 | 0.416 |  |
| T16288C | 0.00 | 0 | 0.52 | 2 | 0.549 |  |
| C16290A | 0.00 | 0 | 0.26 | 1 | 1.000 |  |
| C16290T | 0.50 | 1 | 0.52 | 2 | 1.000 |  |
| C16291T | 2.50 | 5 | 2.86 | 11 | 0.802 |  |
| C16292T | 1.50 | 3 | 2.08 | 8 | 0.757 |  |
| A16293G | 1.50 | 3 | 0.52 | 2 | 0.344 |  |
| C16294T | 11.50 | 23 | 9.61 | 37 | 0.475 |  |
| C16295T | 0.50 | 1 | 1.04 | 4 | 0.665 |  |
| C16296T | 6.00 | 12 | 6.49 | 25 | 0.816 |  |
| T16298C | 2.50 | 5 | 5.97 | 23 | 0.062 |  |
| A16299G | 0.50 | 1 | 0.26 | 1 | 1.000 |  |
| A16300G | 0.50 | 1 | 0.26 | 1 | 1.000 |  |
| T16304C | 8.50 | 17 | 9.87 | 38 | 0.590 |  |
| A16309G | 1.00 | 2 | 0.78 | 3 | 1.000 |  |
| T16311C | 14.00 | 28 | 9.87 | 38 | 0.134 |  |
| T16311TC^f^ | 0.50 | 1 | 0.00 | 0 | 0.342 |  |
| A16316G | 1.50 | 3 | 1.82 | 7 | 1.000 |  |
| A16318G | 0.00 | 0 | 0.26 | 1 | 1.000 |  |
| A16318T | 1.00 | 2 | 0.52 | 2 | 0.609 |  |
| G16319A | 2.50 | 5 | 3.64 | 14 | 0.462 |  |
| C16320T | 1.50 | 3 | 0.52 | 2 | 0.344 |  |
| T16324C | 2.50 | 5 | 0.26 | 1 | 0.020 | 9.85 (1.1-84.9) |
| T16325C | 0.50 | 1 | 1.82 | 7 | 0.275 |  |
| C16327T | 0.00 | 0 | 1.04 | 4 | 0.305 |  |
| C16328A | 0.50 | 1 | 0.00 | 0 | 0.342 |  |
| T16342C | 0.50 | 1 | 0.26 | 1 | 1.000 |  |
| A16343G | 0.50 | 1 | 1.30 | 5 | 0.669 |  |
| C16344T | 0.50 | 1 | 0.00 | 0 | 0.342 |  |
| T16352C | 1.00 | 2 | 1.04 | 4 | 1.000 |  |
| C16353T | 0.00 | 0 | 0.26 | 1 | 1.000 |  |
| C16354T | 1.00 | 2 | 1.56 | 6 | 0.722 |  |
| C16355T | 1.00 | 2 | 0.52 | 2 | 0.609 |  |
| T16356C | 5.50 | 11 | 7.27 | 28 | 0.415 |  |
| T16357C | 0.50 | 1 | 0.52 | 2 | 1.000 |  |
| C16360T | 0.50 | 1 | 0.26 | 1 | 1.000 |  |
| T16362C | 7.50 | 15 | 6.49 | 25 | 0.647 |  |
| C16366T | 0.50 | 1 | 0.00 | 0 | 0.342 |  |
| T16368C | 0.00 | 0 | 0.52 | 2 | 0.549 |  |
| C16380T | 0.00 | 0 | 0.26 | 1 | 1.000 |  |
| G16390A | 1.00 | 2 | 1.30 | 5 | 1.000 |  |
| G16391A | 2.00 | 4 | 0.78 | 3 | 0.238 |  |
| A16399G | 6.00 | 12 | 2.86 | 11 | 0.064 |  |
| C16400T | 0.50 | 1 | 0.00 | 0 | 0.342 |  |
| T16422C | 0.00 | 0 | 0.26 | 1 | 1.000 |  |
| G16438A | 0.00 | 0 | 0.78 | 3 | 0.555 |  |
| A16463G | 0.50 | 1 | 1.30 | 5 | 0.669 |  |
| C16465T | 0.00 | 0 | 0.26 | 1 | 1.000 |  |
| A16482G | 0.50 | 1 | 0.26 | 1 | 1.000 |  |
| A16497G | 0.50 | 1 | 0.26 | 1 | 1.000 |  |
| T16506C^f^ | 0.00 | 0 | 0.26 | 1 | 1.000 |  |
| T16519C | 63.00 | 126 | 65.45 | 252 | 0.556 |  |
| G16526A | 0.50 | 1 | 2.08 | 8 | 0.176 |  |
| C16527T | 0.50 | 1 | 0.52 | 2 | 1.000 |  |
| C41T | 0.00 | 0 | 0.26 | 1 | 1.000 |  |
| T55C | 0.50 | 1 | 0.00 | 0 | 0.342 |  |
| A56InsT^f^ | 0.50 | 1 | 0.00 | 0 | 0.342 |  |
| T57C | 0.50 | 1 | 0.00 | 0 | 0.342 |  |
| C64T | 0.50 | 1 | 0.26 | 1 | 1.000 |  |
| G71A | 0.00 | 0 | 0.52 | 2 | 0.549 |  |
| T72C | 2.00 | 4 | 3.12 | 12 | 0.432 |  |
| A73G | 62.50 | 125 | 51.43 | 198 | 0.011 | 1.57 (1.1-2.2) |
| T74G^f^ | 0.00 | 0 | 0.26 | 1 | 1.000 |  |
| A93G | 3.50 | 7 | 1.56 | 6 | 0.146 |  |
| C114T | 0.50 | 1 | 0.00 | 0 | 0.342 |  |
| T119C | 0.00 | 0 | 0.52 | 2 | 0.549 |  |
| G143A | 0.00 | 0 | 1.04 | 4 | 0.305 |  |
| T146C | 12.50 | 25 | 8.05 | 31 | 0.083 |  |
| C150T | 12.50 | 25 | 10.13 | 39 | 0.384 |  |
| C151T | 1.50 | 3 | 1.04 | 4 | 0.695 |  |
| T152C | 28.50 | 57 | 20.78 | 80 | 0.036 | 1.52 (1.0-2.3) |
| A153G | 3.50 | 7 | 2.34 | 9 | 0.414 |  |
| T159C | 0.00 | 0 | 0.52 | 2 | 0.549 |  |
| C182T | 0.50 | 1 | 0.00 | 0 | 0.342 |  |
| A183G | 0.50 | 1 | 0.52 | 2 | 1.000 |  |
| G185A | 8.00 | 16 | 5.71 | 22 | 0.287 |  |
| C186A | 0.00 | 0 | 0.26 | 1 | 1.000 |  |
| A188G | 1.00 | 2 | 1.56 | 6 | 0.722 |  |
| A189G | 1.50 | 3 | 3.64 | 14 | 0.145 |  |
| A193G | 0.00 | 0 | 0.52 | 2 | 0.549 |  |
| C194T | 1.50 | 3 | 2.60 | 10 | 0.558 |  |
| T195C | 18.50 | 37 | 17.92 | 69 | 0.863 |  |
| T196C | 0.00 | 0 | 0.26 | 1 | 1.000 |  |
| C198T | 1.00 | 2 | 0.26 | 1 | 0.270 |  |
| T199C | 3.00 | 6 | 2.86 | 11 | 0.922 |  |
| A200G | 1.00 | 2 | 1.82 | 7 | 0.725 |  |
| G203A | 0.50 | 1 | 0.00 | 0 | 0.342 |  |
| T204C | 3.00 | 6 | 3.38 | 13 | 0.807 |  |
| G207A | 2.00 | 4 | 3.64 | 14 | 0.277 |  |
| A210G | 0.00 | 0 | 0.78 | 3 | 0.555 |  |
| A214G | 0.50 | 1 | 0.26 | 1 | 1.000 |  |
| A215G | 5.00 | 10 | 2.86 | 11 | 0.186 |  |
| T217C | 1.00 | 2 | 0.78 | 3 | 1.000 |  |
| C222T | 0.50 | 1 | 0.00 | 0 | 0.342 |  |
| G225A | 2.00 | 4 | 2.08 | 8 | 1.000 |  |
| T226C | 0.50 | 1 | 0.78 | 3 | 1.000 |  |
| A227G | 1.50 | 3 | 0.78 | 3 | 0.416 |  |
| G228A | 8.50 | 17 | 5.97 | 23 | 0.251 |  |
| A235G | 0.00 | 0 | 0.26 | 1 | 1.000 |  |
| T236C | 0.00 | 0 | 0.26 | 1 | 1.000 |  |
| T239C | 1.00 | 2 | 0.78 | 3 | 1.000 |  |
| C242T | 2.50 | 5 | 0.52 | 2 | 0.049 | 4.91 (0.9-25.5) |
| G247A | 0.00 | 0 | 0.26 | 1 | 1.000 |  |
| G247Del | 0.50 | 1 | 0.00 | 0 | 0.342 |  |
| A248G | 0.50 | 1 | 0.00 | 0 | 0.342 |  |
| A248Del | 0.00 | 0 | 0.26 | 1 | 1.000 |  |
| A249G | 0.00 | 0 | 0.26 | 1 | 1.000 |  |
| A249Del | 0.00 | 0 | 0.26 | 1 | 1.000 |  |
| T250C | 2.00 | 4 | 0.78 | 3 | 0.238 |  |
| A257G | 0.50 | 1 | 0.00 | 0 | 0.342 |  |
| A263G | 98.50 | 197 | 98.44 | 379 | 1.000 |  |
| T279C | 1.00 | 2 | 0.00 | 0 | 0.116 |  |
| T282C | 0.50 | 1 | 0.00 | 0 | 0.342 |  |
| C285T | 0.00 | 0 | 0.52 | 2 | 0.549 |  |
| T292C | 0.50 | 1 | 0.00 | 0 | 0.342 |  |
| T294C | 0.00 | 0 | 0.26 | 1 | 1.000 |  |
| C295T | 16.50 | 33 | 10.39 | 40 | 0.034 | 1.70 (1.0-2.8) |
| A302InsC | 43.50 | 87 | 36.62 | 141 | 0.106 |  |
| A302InsCC | 9.00 | 18 | 13.25 | 51 | 0.131 |  |
| T310C | 2.00 | 4 | 1.56 | 6 | 0.742 |  |
| T310InsC | 94.50 | 189 | 94.03 | 362 | 0.816 |  |
| T310InsCC | 0.00 | 0 | 0.78 | 3 | 0.555 |  |
| T310InsTC | 3.50 | 7 | 2.60 | 10 | 0.538 |  |
| G316A | 0.50 | 1 | 0.00 | 0 | 0.342 |  |
| T318C | 0.50 | 1 | 0.00 | 0 | 0.342 |  |
| T319C | 5.00 | 10 | 2.86 | 11 | 0.186 |  |
| A337G | 0.00 | 0 | 0.26 | 1 | 1.000 |  |
| C338T | 0.50 | 1 | 0.00 | 0 | 0.342 |  |
| C340T | 1.00 | 2 | 0.26 | 1 | 0.270 |  |
| A368G | 0.50 | 1 | 0.00 | 0 | 0.342 |  |
| A374G | 0.50 | 1 | 0.00 | 0 | 0.342 |  |
| A384G | 0.00 | 0 | 0.52 | 2 | 0.549 |  |
| A385G | 0.00 | 0 | 0.52 | 2 | 0.549 |  |
| T408A | 0.50 | 1 | 0.78 | 3 | 1.000 |  |
| A444G | 0.00 | 0 | 0.26 | 1 | 1.000 |  |
| A451InsT^f^ | 0.50 | 1 | 0.26 | 1 | 1.000 |  |
| T452Del | 0.00 | 0 | 0.26 | 1 | 1.000 |  |
| T453C | 0.50 | 1 | 0.00 | 0 | 0.342 |  |
| C456T | 2.00 | 4 | 3.90 | 15 | 0.220 |  |
| C458T | 0.00 | 0 | 0.26 | 1 | 1.000 |  |
| C462T | 11.00 | 22 | 6.75 | 26 | 0.076 |  |
| C469A | 0.00 | 0 | 0.26 | 1 | 1.000 |  |
| A472G^f^ | 0.00 | 0 | 0.26 | 1 | 1.000 |  |
| T477C | 2.00 | 4 | 3.38 | 13 | 0.347 |  |
| T482C | 0.50 | 1 | 1.30 | 5 | 0.669 |  |
| A487T^f^ | 0.50 | 1 | 0.00 | 0 | 0.342 |  |
| T489C | 16.50 | 33 | 10.91 | 42 | 0.055 |  |
| T489G^f^ | 0.50 | 1 | 0.00 | 0 | 0.342 |  |
| C494Del | 0.50 | 1 | 0.26 | 1 | 1.000 |  |
| C497T | 4.00 | 8 | 1.30 | 5 | 0.071 |  |
| G499A | 4.00 | 8 | 6.23 | 24 | 0.260 |  |
| T504C | 0.00 | 0 | 0.26 | 1 | 1.000 |  |
| C506T | 1.00 | 2 | 0.00 | 0 | 0.116 |  |
| A508G | 1.00 | 2 | 0.78 | 3 | 1.000 |  |
| G513A | 5.00 | 10 | 2.86 | 11 | 0.186 |  |
| G513InsCA | 4.50 | 9 | 5.97 | 23 | 0.457 |  |
| G513InsCACA | 1.50 | 3 | 3.38 | 13 | 0.187 |  |
| G513InsCACACA | 0.50 | 1 | 0.78 | 3 | 1.000 |  |
| G513InsCACACACA | 0.00 | 0 | 0.26 | 1 | 1.000 |  |
| C514T^f^ | 0.50 | 1 | 0.00 | 0 | 0.342 |  |
| CA514/515Del | 10.50 | 21 | 10.13 | 39 | 0.889 |  |
| C516T | 0.00 | 0 | 0.26 | 1 | 1.000 |  |
| A517G | 0.00 | 0 | 0.26 | 1 | 1.000 |  |
| A533G | 0.00 | 0 | 0.52 | 2 | 0.549 |  |
| C535T | 0.50 | 1 | 0.00 | 0 | 0.342 |  |
| A554C^f^ | 0.00 | 0 | 0.26 | 1 | 1.000 |  |
| A567InsC | 2.50 | 5 | 0.00 | 0 | 0.005 |  |
| A567InsCC | 1.00 | 2 | 0.26 | 1 | 0.270 |  |
| A567InsCCC | 0.50 | 1 | 0.00 | 0 | 0.342 |  |
| C568T | 0.50 | 1 | 0.52 | 2 | 1.000 |  |
| C569A^f^ | 0.50 | 1 | 0.00 | 0 | 0.342 |  |

^a^mtDNA = mitochondrial DNA.

^b^CNV = choroidal neovascularisation.

^c^n: number of individuals with the respective polymorphism.

^d^P-value: Pearson chi-square or Fisher’s exact test.

^e^CI = confidence interval.

^f^Polymorphisms not listed in MITOMAP and the Human Mitochondrial Genome Database.
