# Supplementary material for: Mitochondrial Haplogroups and Control Region Polymorphisms in Age-Related Macular Degeneration: A Case-Control Study
Source: PLoS One. 2012 Feb 13;7(2):e30874. doi: 10.1371/journal.pone.0030874 (PMC3278404; doi:10.1371/journal.pone.0030874)
Supplement: Table S2 — Frequencies (%) of control region (CR) polymorphisms in patients with dry AMD and in controls as well as the corresponding odds ratios and 95% confidence intervals. (DOC) [file pone.0030874.s002.doc]

**Table S2.** Frequencies (%) of control region (CR) polymorphisms in patients with dry AMD and in controls as well as the corresponding odds ratios and 95% confidence intervals.

| Polymorphism in mtDNA^a^ control region | Frequency in patients with dry AMD^b^ | n^c^ | Frequency in control group | n^c^ | P-value^d^ | Odds ratio (95% CI^e^) |
| --- | --- | --- | --- | --- | --- | --- |
|  | n=66 |  | n=385 |  |  |  |
| T16045C^f^ | 0.00 | 0 | 0.26 | 1 | 1.000 |  |
| A16051G | 3.03 | 2 | 1.82 | 7 | 0.626 |  |
| C16067T | 0.00 | 0 | 0.52 | 2 | 1.000 |  |
| C16069T | 9.09 | 6 | 10.13 | 39 | 0.795 |  |
| C16072T | 0.00 | 0 | 0.26 | 1 | 1.000 |  |
| A16080G | 0.00 | 0 | 0.52 | 2 | 1.000 |  |
| T16086C | 1.52 | 1 | 0.78 | 3 | 0.470 |  |
| T16092C | 3.03 | 2 | 0.52 | 2 | 0.104 |  |
| T16093C | 7.58 | 5 | 8.31 | 32 | 0.840 |  |
| C16111T | 0.00 | 0 | 0.52 | 2 | 1.000 |  |
| C16114A | 0.00 | 0 | 0.26 | 1 | 1.000 |  |
| T16124C | 0.00 | 0 | 0.78 | 3 | 1.000 |  |
| T16126C | 16.67 | 11 | 18.70 | 72 | 0.694 |  |
| G16129A | 3.03 | 2 | 4.68 | 18 | 0.752 |  |
| G16129C | 3.03 | 2 | 0.78 | 3 | 0.157 |  |
| C16134T | 1.52 | 1 | 0.78 | 3 | 0.470 |  |
| G16145A | 6.06 | 4 | 4.16 | 16 | 0.514 |  |
| C16148T | 1.52 | 1 | 1.04 | 4 | 0.548 |  |
| C16150T | 1.52 | 1 | 0.00 | 0 | 0.146 |  |
| G16153A | 1.52 | 1 | 0.26 | 1 | 0.272 |  |
| A16162G | 3.03 | 2 | 3.38 | 13 | 1.000 |  |
| A16162C | 0.00 | 0 | 0.26 | 1 | 1.000 |  |
| A16163G | 3.03 | 2 | 1.04 | 4 | 0.214 |  |
| A16166G | 0.00 | 0 | 0.26 | 1 | 1.000 |  |
| C16168T | 0.00 | 0 | 0.26 | 1 | 1.000 |  |
| C16169T | 1.52 | 1 | 1.04 | 4 | 0.548 |  |
| A16170G | 0.00 | 0 | 0.26 | 1 | 1.000 |  |
| T16172C | 1.52 | 1 | 1.04 | 4 | 0.548 |  |
| C16173T | 0.00 | 0 | 0.26 | 1 | 1.000 |  |
| C16174T | 0.50 | 1 | 0.26 | 1 | 1.000 |  |
| C16176G | 0.00 | 0 | 0.26 | 1 | 1.000 |  |
| C16179T | 4.55 | 3 | 3.64 | 14 | 0.725 |  |
| A16182G | 0.00 | 0 | 0.26 | 1 | 1.000 |  |
| A16182C | 3.03 | 2 | 2.08 | 8 | 0.645 |  |
| A16183C | 7.58 | 5 | 3.90 | 15 | 0.192 |  |
| C16184T | 0.00 | 0 | 0.52 | 2 | 1.000 |  |
| C16184Del | 1.52 | 1 | 0.00 | 0 | 0.146 |  |
| C16185T | 1.52 | 1 | 0.26 | 1 | 0.272 |  |
| C16186T | 3.03 | 2 | 1.04 | 4 | 0.214 |  |
| C16188T | 1.52 | 1 | 0.52 | 2 | 0.379 |  |
| T16189A | 0.00 | 0 | 0.26 | 1 | 1.000 |  |
| T16189C | 13.64 | 9 | 11.17 | 43 | 0.562 |  |
| T16189InsCC | 1.52 | 1 | 0.00 | 0 | 0.146 |  |
| C16192T | 9.09 | 6 | 4.16 | 16 | 0.114 |  |
| C16193T | 1.52 | 1 | 1.56 | 6 | 1.000 |  |
| C16201T | 1.52 | 1 | 0.52 | 2 | 0.379 |  |
| A16207G | 1.52 | 1 | 0.26 | 1 | 0.272 |  |
| A16212G | 0.00 | 0 | 0.78 | 3 | 1.000 |  |
| G16213A | 1.52 | 1 | 0.78 | 3 | 0.470 |  |
| T16217C | 1.52 | 1 | 0.00 | 0 | 0.146 |  |
| C16218T | 1.52 | 1 | 0.26 | 1 | 0.272 |  |
| C16221T | 0.00 | 0 | 0.78 | 3 | 1.000 |  |
| C16222T | 1.52 | 1 | 1.30 | 5 | 1.000 |  |
| C16223T | 12.12 | 8 | 7.01 | 27 | 0.152 |  |
| T16224C | 4.55 | 3 | 4.94 | 19 | 1.000 |  |
| T16231C | 3.03 | 2 | 3.12 | 12 | 1.000 |  |
| C16234T | 0.00 | 0 | 1.30 | 5 | 1.000 |  |
| A16235G | 0.00 | 0 | 0.52 | 2 | 1.000 |  |
| C16239T | 0.00 | 0 | 1.04 | 4 | 1.000 |  |
| A16241G | 1.52 | 1 | 0.00 | 0 | 0.146 |  |
| C16242T | 0.00 | 0 | 0.52 | 2 | 1.000 |  |
| T16243C | 0.00 | 0 | 0.26 | 1 | 1.000 |  |
| G16244A | 0.00 | 0 | 0.26 | 1 | 1.000 |  |
| C16245T | 0.00 | 0 | 0.26 | 1 | 1.000 |  |
| C16248T | 0.00 | 0 | 0.26 | 1 | 1.000 |  |
| T16249C | 0.00 | 0 | 1.04 | 4 | 1.000 |  |
| G16255A | 1.52 | 1 | 1.04 | 4 | 0.548 |  |
| C16256T | 3.03 | 2 | 4.94 | 19 | 0.753 |  |
| A16258T | 0.00 | 0 | 0.26 | 1 | 1.000 |  |
| A16258C | 3.03 | 2 | 1.56 | 6 | 0.331 |  |
| C16259T | 1.52 | 1 | 0.00 | 0 | 0.146 |  |
| C16261T | 6.06 | 4 | 3.90 | 15 | 0.502 |  |
| T16263C | 0.00 | 0 | 0.78 | 3 | 1.000 |  |
| A16265G | 3.03 | 2 | 1.04 | 4 | 0.214 |  |
| C16266T | 0.00 | 0 | 0.52 | 2 | 1.000 |  |
| C16268T | 0.00 | 0 | 0.26 | 1 | 1.000 |  |
| A16269G | 0.00 | 0 | 0.26 | 1 | 1.000 |  |
| C16270T | 3.03 | 2 | 5.71 | 22 | 0.555 |  |
| C16270G^f^ | 1.52 | 1 | 0.00 | 0 | 0.146 |  |
| T16271C | 0.00 | 0 | 0.78 | 3 | 1.000 |  |
| G16273A | 1.52 | 1 | 0.00 | 0 | 0.146 |  |
| G16274A | 0.00 | 0 | 0.78 | 3 | 1.000 |  |
| C16278T | 4.55 | 3 | 3.12 | 12 | 0.469 |  |
| A16283T | 0.00 | 0 | 0.26 | 1 | 1.000 |  |
| C16287T | 0.00 | 0 | 0.78 | 3 | 1.000 |  |
| T16288C | 0.00 | 0 | 0.52 | 2 | 1.000 |  |
| C16290A | 0.00 | 0 | 0.26 | 1 | 1.000 |  |
| C16290T | 0.00 | 0 | 0.52 | 2 | 1.000 |  |
| C16291T | 1.52 | 1 | 2.86 | 11 | 1.000 |  |
| C16292T | 6.06 | 4 | 2.08 | 8 | 0.083 |  |
| A16293G | 1.52 | 1 | 0.52 | 2 | 0.379 |  |
| C16294T | 9.09 | 6 | 9.61 | 37 | 0.894 |  |
| C16295T | 0.00 | 0 | 1.04 | 4 | 1.000 |  |
| C16296T | 3.03 | 2 | 6.49 | 25 | 0.401 |  |
| T16298C | 6.06 | 4 | 5.97 | 23 | 1.000 |  |
| A16299G | 1.52 | 1 | 0.26 | 1 | 0.272 |  |
| A16300G | 1.52 | 1 | 0.26 | 1 | 0.272 |  |
| C16301T | 1.52 | 1 | 0.00 | 0 | 0.146 |  |
| T16304C | 9.09 | 6 | 9.87 | 38 | 0.844 |  |
| A16309G | 0.00 | 0 | 0.78 | 3 | 1.000 |  |
| T16311C | 12.12 | 8 | 9.87 | 38 | 0.577 |  |
| A16316G | 1.52 | 1 | 1.82 | 7 | 1.000 |  |
| A16318G | 0.00 | 0 | 0.26 | 1 | 1.000 |  |
| A16318T | 0.00 | 0 | 0.52 | 2 | 1.000 |  |
| G16319A | 1.52 | 1 | 3.64 | 14 | 0.708 |  |
| C16320T | 1.52 | 1 | 0.52 | 2 | 0.379 |  |
| T16324C | 0.00 | 0 | 0.26 | 1 | 1.000 |  |
| T16325C | 4.55 | 3 | 1.82 | 7 | 0.168 |  |
| C16327T | 0.00 | 0 | 1.04 | 4 | 1.000 |  |
| T16342C | 0.00 | 0 | 0.26 | 1 | 1.000 |  |
| A16343G | 1.52 | 1 | 1.30 | 5 | 1.000 |  |
| T16352C | 1.52 | 1 | 1.04 | 4 | 0.548 |  |
| C16353T | 0.00 | 0 | 0.26 | 1 | 1.000 |  |
| C16354T | 3.03 | 2 | 1.56 | 6 | 0.331 |  |
| C16355T | 1.52 | 1 | 0.52 | 2 | 0.379 |  |
| T16356C | 7.58 | 5 | 7.27 | 28 | 1.000 |  |
| T16357C | 0.00 | 0 | 0.52 | 2 | 1.000 |  |
| C16360T | 0.00 | 0 | 0.26 | 1 | 1.000 |  |
| T16362C | 6.06 | 4 | 6.49 | 25 | 1.000 |  |
| T16368C | 0.00 | 0 | 0.52 | 2 | 1.000 |  |
| C16380T | 0.00 | 0 | 0.26 | 1 | 1.000 |  |
| G16390A | 3.03 | 2 | 1.30 | 5 | 0.273 |  |
| G16391A | 0.00 | 0 | 0.78 | 3 | 1.000 |  |
| G16398A | 1.52 | 1 | 0.00 | 0 | 0.146 |  |
| A16399G | 0.00 | 0 | 2.86 | 11 | 0.380 |  |
| T16422C | 0.00 | 0 | 0.26 | 1 | 1.000 |  |
| G16438A | 0.00 | 0 | 0.78 | 3 | 1.000 |  |
| A16463G | 0.00 | 0 | 1.30 | 5 | 1.000 |  |
| C16465T | 0.00 | 0 | 0.26 | 1 | 1.000 |  |
| A16482G | 1.52 | 1 | 0.26 | 1 | 0.272 |  |
| A16497G | 0.00 | 0 | 0.26 | 1 | 1.000 |  |
| T16506C^f^ | 0.00 | 0 | 0.26 | 1 | 1.000 |  |
| T16519C | 75.76 | 50 | 65.45 | 252 | 0.100 |  |
| G16526A | 0.00 | 0 | 2.08 | 8 | 0.610 |  |
| C16527T | 0.00 | 0 | 0.52 | 2 | 1.000 |  |
| C41T | 0.00 | 0 | 0.26 | 1 | 1.000 |  |
| C64T | 0.00 | 0 | 0.26 | 1 | 1.000 |  |
| G71A | 0.00 | 0 | 0.52 | 2 | 1.000 |  |
| T72C | 3.03 | 2 | 3.12 | 12 | 1.000 |  |
| A73G | 54.55 | 36 | 51.43 | 198 | 0.640 |  |
| T74G^f^ | 0.00 | 0 | 0.26 | 1 | 1.000 |  |
| A93G | 6.06 | 4 | 1.56 | 6 | 0.044 | 4.08 (1.1-14.9) |
| T119C | 0.00 | 0 | 0.52 | 2 | 1.000 |  |
| G143A | 1.52 | 1 | 1.04 | 4 | 0.548 |  |
| T146C | 7.58 | 5 | 8.05 | 31 | 0.895 |  |
| C150T | 12.12 | 8 | 10.13 | 39 | 0.625 |  |
| C151T | 1.52 | 1 | 1.04 | 4 | 0.548 |  |
| T152C | 24.24 | 16 | 20.78 | 80 | 0.525 |  |
| A153G | 3.03 | 2 | 2.34 | 9 | 0.667 |  |
| T159C | 0.00 | 0 | 0.52 | 2 | 1.000 |  |
| A183G | 1.52 | 1 | 0.52 | 2 | 0.379 |  |
| G185A | 1.52 | 1 | 5.71 | 22 | 0.226 |  |
| C186A | 0.00 | 0 | 0.26 | 1 | 1.000 |  |
| A188G | 0.00 | 0 | 1.56 | 6 | 0.599 |  |
| A189G | 9.09 | 6 | 3.64 | 14 | 0.096 |  |
| A193G | 0.00 | 0 | 0.52 | 2 | 1.000 |  |
| C194T | 3.03 | 2 | 2.60 | 10 | 0.691 |  |
| T195C | 33.33 | 22 | 17.92 | 69 | 0.004 | 2.29 (1.3-4.1) |
| T196C | 0.00 | 0 | 0.26 | 1 | 1.000 |  |
| C198T | 0.00 | 0 | 0.26 | 1 | 1.000 |  |
| T199C | 0.00 | 0 | 2.86 | 11 | 0.380 |  |
| A200G | 1.52 | 1 | 1.82 | 7 | 1.000 |  |
| T204C | 7.58 | 5 | 3.38 | 13 | 0.161 |  |
| G207A | 9.09 | 6 | 3.64 | 14 | 0.096 |  |
| A210G | 0.00 | 0 | 0.78 | 3 | 1.000 |  |
| A214G | 0.00 | 0 | 0.26 | 1 | 1.000 |  |
| A215G | 3.03 | 2 | 2.86 | 11 | 1.000 |  |
| T217C | 3.03 | 2 | 0.78 | 3 | 0.157 |  |
| G225A | 4.55 | 3 | 2.08 | 8 | 0.208 |  |
| T226C | 1.52 | 1 | 0.78 | 3 | 0.470 |  |
| A227G | 1.52 | 1 | 0.78 | 3 | 0.470 |  |
| G228A | 0.00 | 0 | 5.97 | 23 | 0.035 |  |
| A235G | 0.00 | 0 | 0.26 | 1 | 1.000 |  |
| T236C | 0.00 | 0 | 0.26 | 1 | 1.000 |  |
| T239C | 3.03 | 2 | 0.78 | 3 | 0.157 |  |
| C242T | 1.52 | 1 | 0.52 | 2 | 0.379 |  |
| G247A | 0.00 | 0 | 0.26 | 1 | 1.000 |  |
| A248Del | 0.00 | 0 | 0.26 | 1 | 1.000 |  |
| A249G | 0.00 | 0 | 0.26 | 1 | 1.000 |  |
| A249Del | 0.00 | 0 | 0.26 | 1 | 1.000 |  |
| T250C | 0.00 | 0 | 0.78 | 3 | 1.000 |  |
| C262T | 1.52 | 1 | 0.00 | 0 | 0.146 |  |
| A263G | 100.00 | 66 | 98.44 | 379 | 0.599 |  |
| C285T | 0.00 | 0 | 0.52 | 2 | 1.000 |  |
| T294C | 0.00 | 0 | 0.26 | 1 | 1.000 |  |
| C295T | 7.58 | 5 | 10.39 | 40 | 0.481 |  |
| A302InsC | 30.30 | 20 | 36.62 | 141 | 0.322 |  |
| A302InsCC | 10.61 | 7 | 13.25 | 51 | 0.554 |  |
| T310C | 0.00 | 0 | 1.56 | 6 | 0.599 |  |
| T310InsC | 93.94 | 62 | 94.03 | 362 | 1.000 |  |
| T310InsCC | 0.00 | 0 | 0.78 | 3 | 1.000 |  |
| T310InsTC | 3.03 | 2 | 2.60 | 10 | 0.691 |  |
| T319C | 3.03 | 2 | 2.86 | 11 | 1.000 |  |
| A337G | 0.00 | 0 | 0.26 | 1 | 1.000 |  |
| C340T | 1.52 | 1 | 0.26 | 1 | 0.272 |  |
| A384G | 0.00 | 0 | 0.52 | 2 | 1.000 |  |
| A385G | 0.00 | 0 | 0.52 | 2 | 1.000 |  |
| T408A | 0.00 | 0 | 0.78 | 3 | 1.000 |  |
| A444G | 0.00 | 0 | 0.26 | 1 | 1.000 |  |
| A451InsT^f^ | 0.00 | 0 | 0.26 | 1 | 1.000 |  |
| T452Del | 0.00 | 0 | 0.26 | 1 | 1.000 |  |
| T453C | 1.52 | 1 | 0.00 | 0 | 0.146 |  |
| C456T | 3.03 | 2 | 3.90 | 15 | 1.000 |  |
| C458T | 0.00 | 0 | 0.26 | 1 | 1.000 |  |
| C462T | 3.03 | 2 | 6.75 | 26 | 0.405 |  |
| C469A | 0.00 | 0 | 0.26 | 1 | 1.000 |  |
| A472G^f^ | 0.00 | 0 | 0.26 | 1 | 1.000 |  |
| T477C | 6.06 | 4 | 3.38 | 13 | 0.292 |  |
| T482C | 0.00 | 0 | 1.30 | 5 | 1.000 |  |
| T489C | 7.58 | 5 | 10.91 | 42 | 0.413 |  |
| C494Del | 0.00 | 0 | 0.26 | 1 | 1.000 |  |
| C497T | 3.03 | 2 | 1.30 | 5 | 0.273 |  |
| G499A | 7.58 | 5 | 6.23 | 24 | 0.596 |  |
| T504C | 0.00 | 0 | 0.26 | 1 | 1.000 |  |
| A508G | 3.03 | 2 | 0.78 | 3 | 0.157 |  |
| G513A | 3.03 | 2 | 2.86 | 11 | 1.000 |  |
| G513InsCA | 6.06 | 4 | 5.97 | 23 | 1.000 |  |
| G513InsCACA | 4.55 | 3 | 3.38 | 13 | 0.715 |  |
| G513InsCACACA | 1.52 | 1 | 0.78 | 3 | 0.470 |  |
| G513InsCACACACA | 0.00 | 0 | 0.26 | 1 | 1.000 |  |
| CA514/515Del | 7.58 | 5 | 10.13 | 39 | 0.518 |  |
| C516T | 1.52 | 1 | 0.26 | 1 | 0.272 |  |
| A517G | 0.00 | 0 | 0.26 | 1 | 1.000 |  |
| A533G | 0.00 | 0 | 0.52 | 2 | 1.000 |  |
| A554C^f^ | 0.00 | 0 | 0.26 | 1 | 1.000 |  |
| A567InsCC | 0.00 | 0 | 0.26 | 1 | 1.000 |  |
| C568T | 0.00 | 0 | 0.52 | 2 | 1.000 |  |
| C569A^f^ | 1.52 | 1 | 0.00 | 0 | 0.146 |  |

^a^mtDNA = mitochondrial DNA.

^b^AMD = age-related macular degeneration.

^c^n: number of individuals with the respective polymorphism.

^d^P-value: Pearson chi-square or Fisher’s exact test.

^e^CI = confidence interval.

^f^Polymorphisms not listed in MITOMAP and the Human Mitochondrial Genome Database.
